# Special Issue: The Role of Gut Microbiota in Gastrointestinal Cancers—From Pathogenesis to Therapeutic Perspectives

**DOI:** 10.3390/biomedicines11112950

**Published:** 2023-11-01

**Authors:** Ryota Niikura

**Affiliations:** Gastroenterological Endoscopy, Tokyo Medical University, Tokyo 160-0023, Japan; rniikura@triton.ocn.ne.jp

## 1. Introduction

Associations between the gut microbiota and gastrointestinal carcinogenesis have been intensively studied. Our Special Issue includes three original articles for the upper gut microbiota, as well as one original article and three review articles for the lower gut microbiota.

## 2. Upper Gut Microbiota

Liu and Teng explore the details of the lipopolysaccharide (LPS) structure in *Helicobacter pylori* (*H. pylori*), focusing on the genes HP0044 and HP1275 [1]. Their findings suggest that mutations in these genes hinder the biosynthesis of fucose—a crucial LPS component—and alter the entire LPS structure and function. These mutations also influence the sorting of virulence factors such as CagA and VacA into outer membrane vesicles (OMVs), pointing to a deep connection of these genes with bacterial pathogenicity. This perspective into GMD and PMM proteins might lay the foundation for more specific drug developments against *H. pylori* infections.

Son and Lee contribute to our knowledge by analyzing the metabolite profile of the gastric mucosa in patients with different *H. pylori* infection statuses [2]. Their nontargeted approach identified 28 distinct metabolites that displayed varied concentrations based on the patients’ infection history. For instance, the elevated levels of certain amino acids and sugars in the noninfected group might imply their protective function against gastric changes. Moreover, the links observed between certain metabolites and gastric atrophy or intestinal metaplasia highlight the bacterium’s comprehensive metabolic impact.

Junya Arai highlights the subtle contrasts between gastric cancers occurring within the setting of autoimmune gastritis (AIG) and those linked to *H. pylori* infections [3]. The marked expression of MUC5AC and MUC6, as well as variations in immune cell infiltration, suggest a unique tumor environment in AIG-associated cancers. Additionally, the different gastric mucosal microbiota, especially the dominance of Bacillus and *Streptococcus* spp., highlight the specific microbes that are evident in AIG patients.

## 3. Lower Gut Microbiota

In a review by Ali Mohamed and Harry Menon [4], they present a comprehensive analysis of the gut microbiome’s role in colon cancer and its impact on chemotherapy outcomes. This insightful review sheds light on the current understanding of the microbiome in normal colon epithelia and sporadic colon cancer. It emphasizes the relevance of the intestinal microbiome in influencing both the efficacy and toxicity of chemotherapy. Furthermore, it delves into the consequences of antibiotic use on the gut microbiome and its association with colon cancer.

In a parallel review by Kevin M. Tourelle and Sebastien Boutin [5], the spotlight is directed toward the impact of the gut microbiome on gastrointestinal carcinoma, with a particular focus on colorectal cancer (CRC) and chemotherapy outcomes. Dysbiosis, characterized by an imbalance in the gut microbiome, can be triggered by various factors, including diet, medications, and specific treatments. *Fusobacterium nucleatum*, a bacterium associated with chemotherapy resistance and cancer recurrence, takes center stage in their analysis. The review highlights the potential of probiotics and certain herbal medicines in reducing chemotherapy-related adverse events and improving patient outcomes. It also emphasizes the influence of surgery for gastrointestinal cancer in directly altering the gut microbiome, giving rise to complications such as anastomotic insufficiencies and abdominal infections.

Focusing on developing analysis methods for the microbiota, Wei and Wu’s research concentrates on the gut ambiance of colorectal patients [6]. They propose an optimistic predictive model for identifying colorectal adenomatous polyps, considering the limitations of the iFOBT. The use of long-read sequencing offers a species-level clarity of the gut microbiota, with certain bacterial groups potentially acting as indicators to differentiate between colorectal cancer and adenomas.

Keita Kouzu and Hironori Tsujimoto contribute a third review [7], exploring the intriguing realm of bacterial translocation in gastrointestinal cancers. The intestinal immune system, which is replete with lymphocytes, plasma cells, and macrophages, plays an important role in maintaining a harmonious function between the host and the diverse array of microorganisms residing in the gut. Bacterial translocation, the movement of bacteria or bacterial components from the gut into the bloodstream or lymphatic system, emerges as a central theme. Recent research has suggested that translocation may involve pathogen-associated molecular patterns (PAMPs) and damage-associated molecular patterns (DAMPs), even in the absence of detectable bacteria in the blood or tissues. Pattern recognition receptors (PRRs), such as toll-like receptors (TLRs), are the sentinels responsible for detecting these patterns and playing a critical role in the immune response.

## 4. Conclusions

For the upper gut microbiota, three original studies emphasize the multifaceted impacts of *H. pylori* on gastric health, from structural changes to metabolic transitions. Furthermore, these studies propose a potential association between new microbiota beyond *H. pylori* and gastric cancer. For the lower gut microbiota, insightful reviews collectively explore the multifaceted relationship between the lower gut microbiome and gastrointestinal cancers. They emphasize the need for a thorough understanding of the microbiota’s role in advanced diagnostic methods, disease progression, and therapy response, offering promising prospects for more effective treatments and improved patient outcomes in the future.

In conclusion, as research in this field continues to advance, the scientific community is prepared to make further progress in comprehending the complexities of gastrointestinal cancers and their intimate connection with the gut microbiome.
